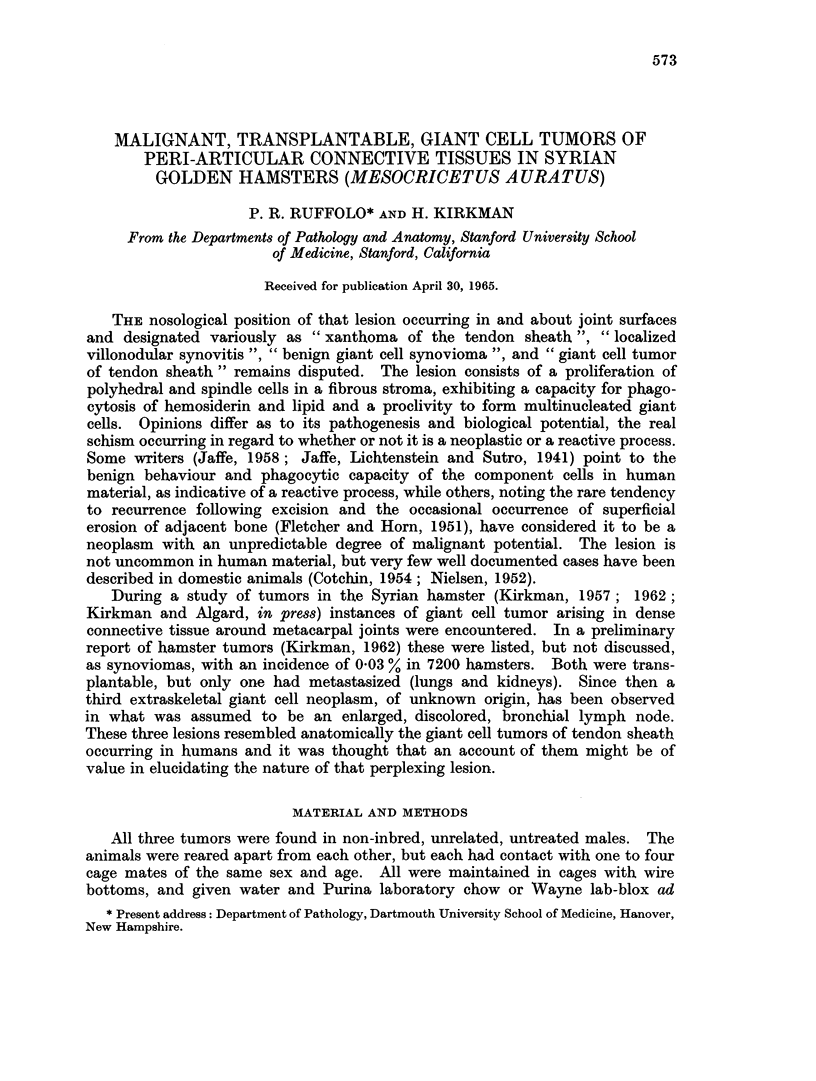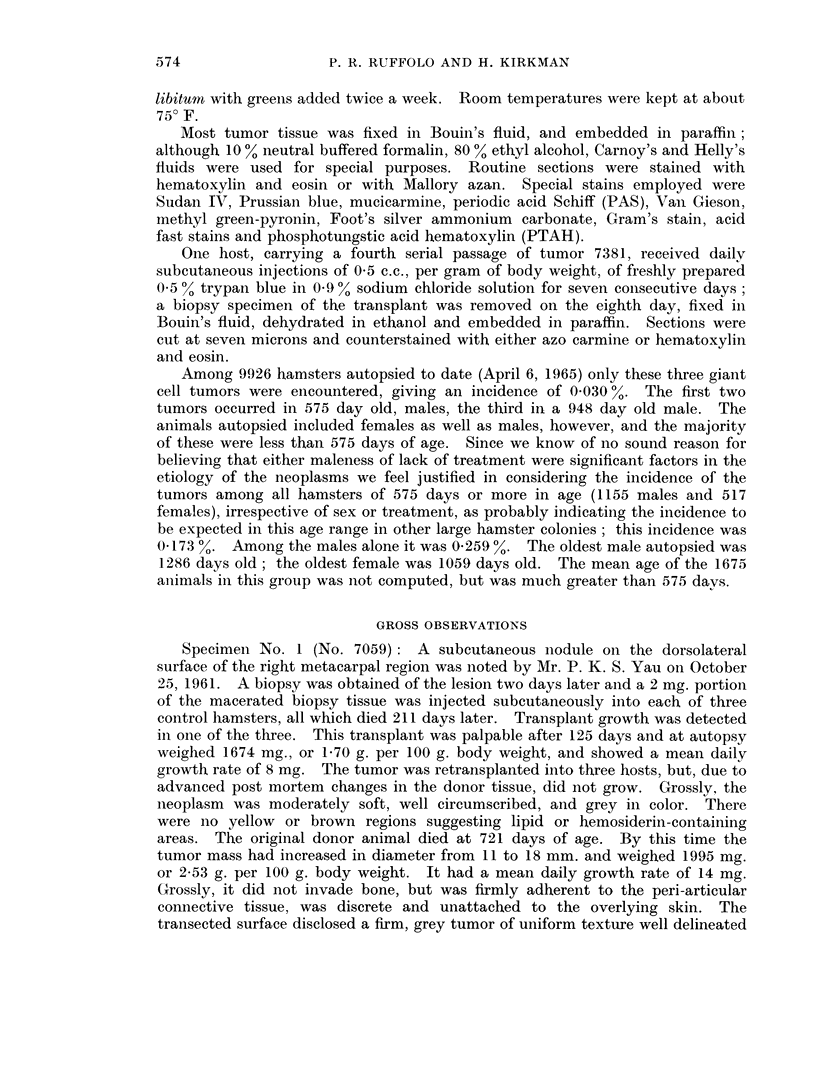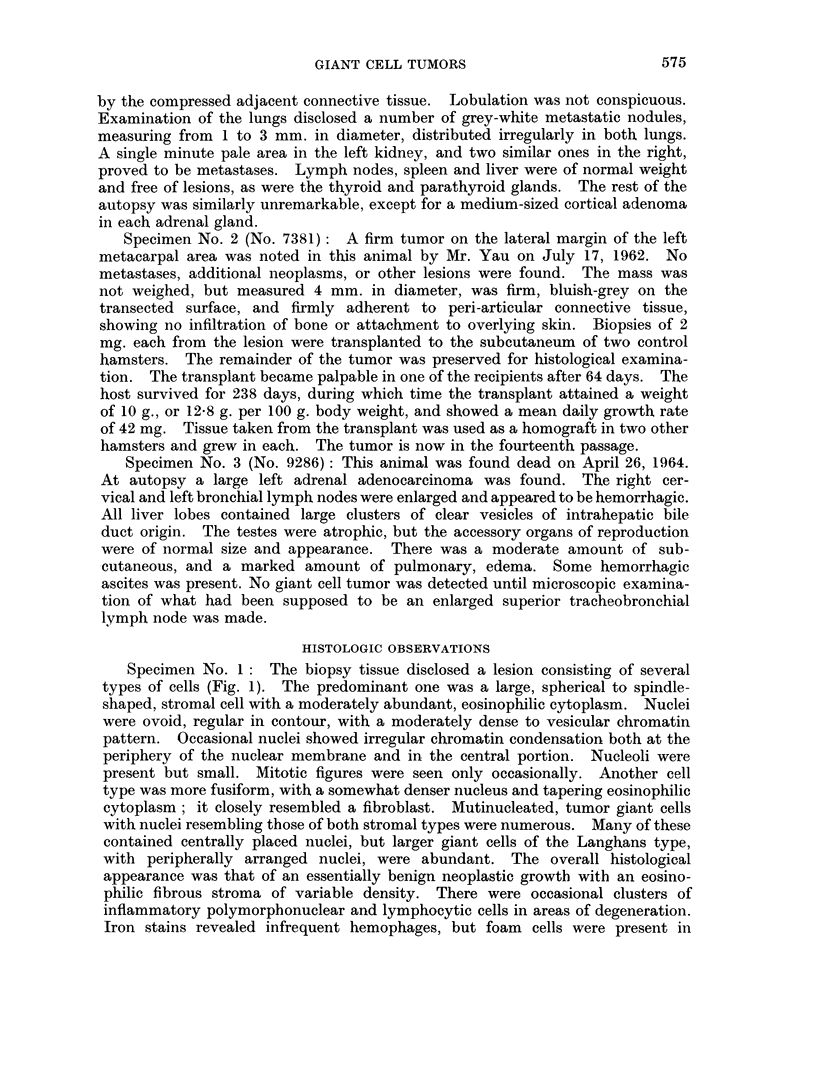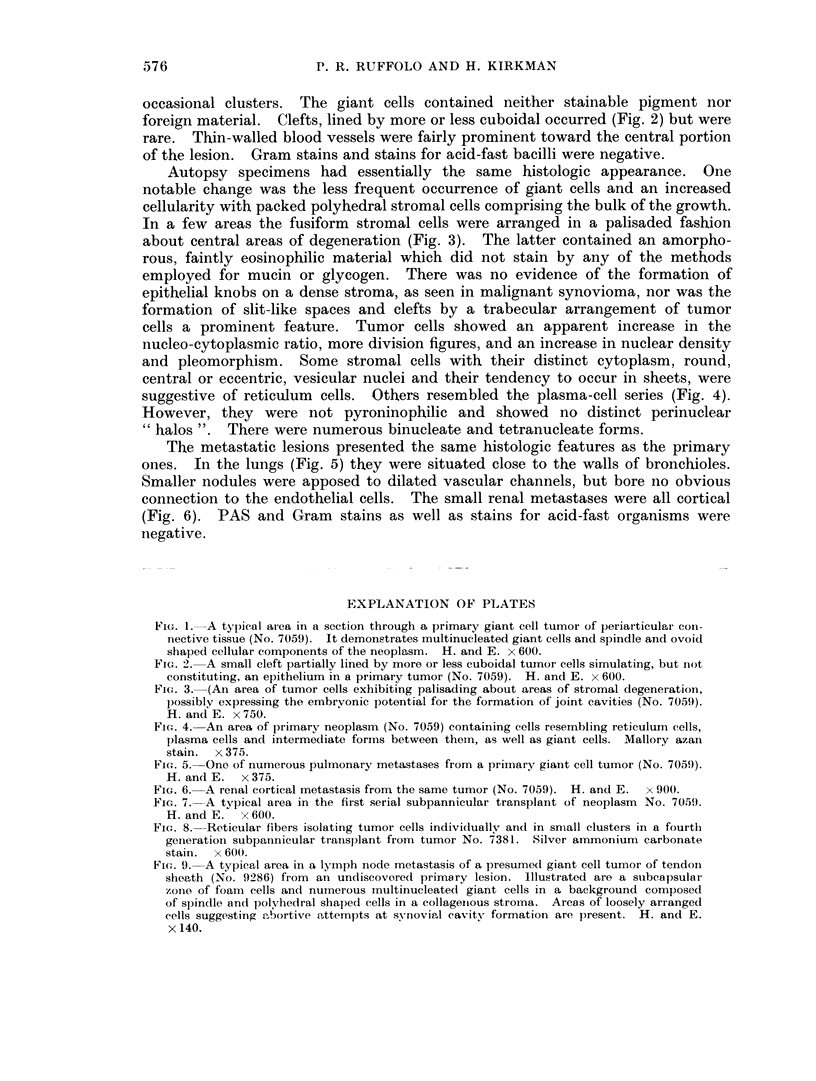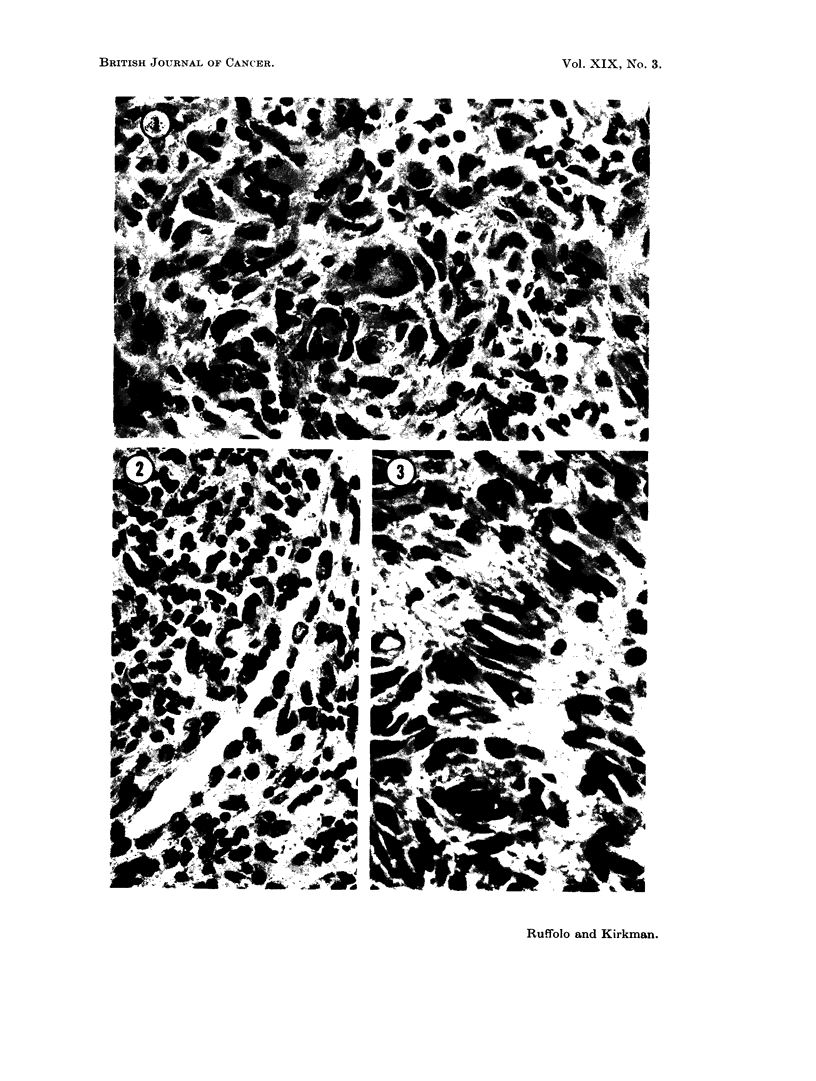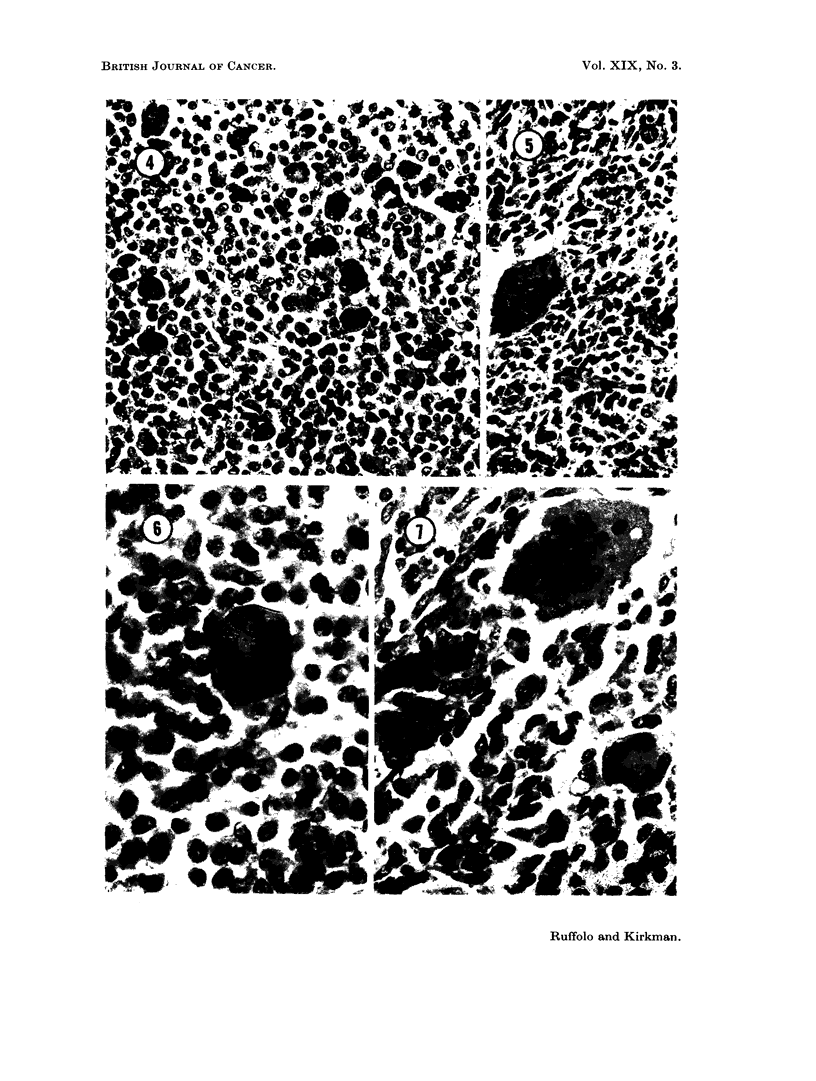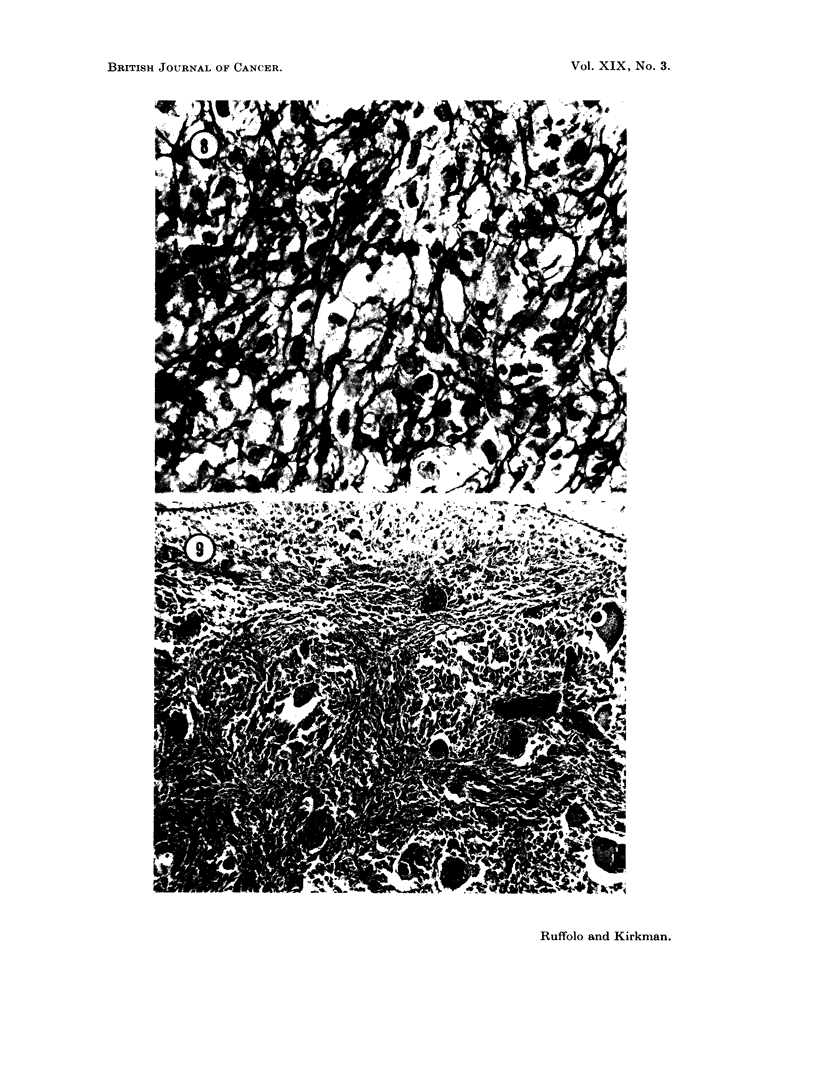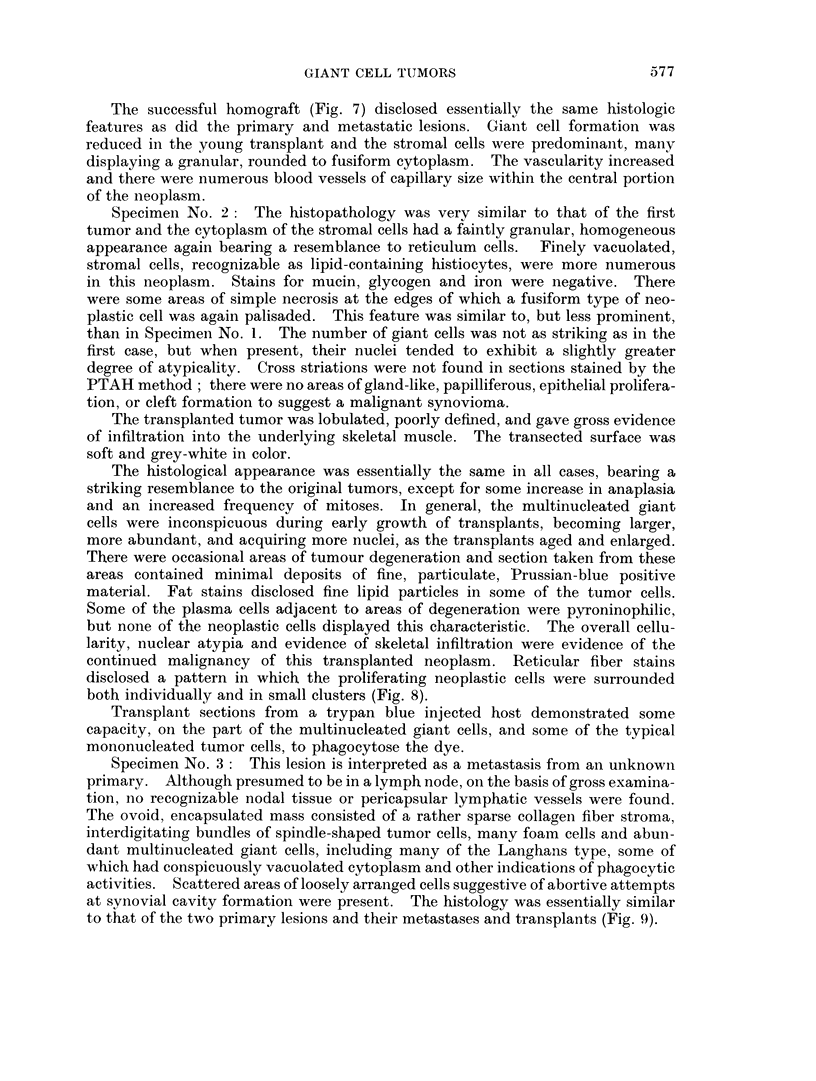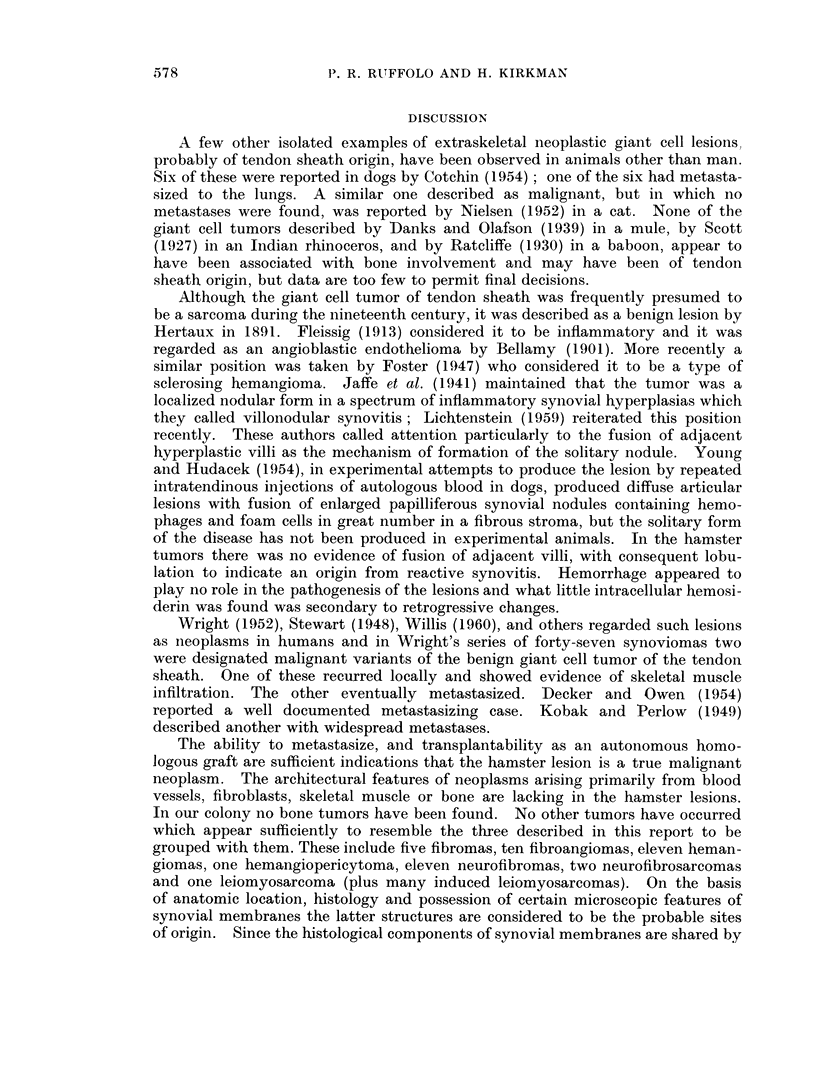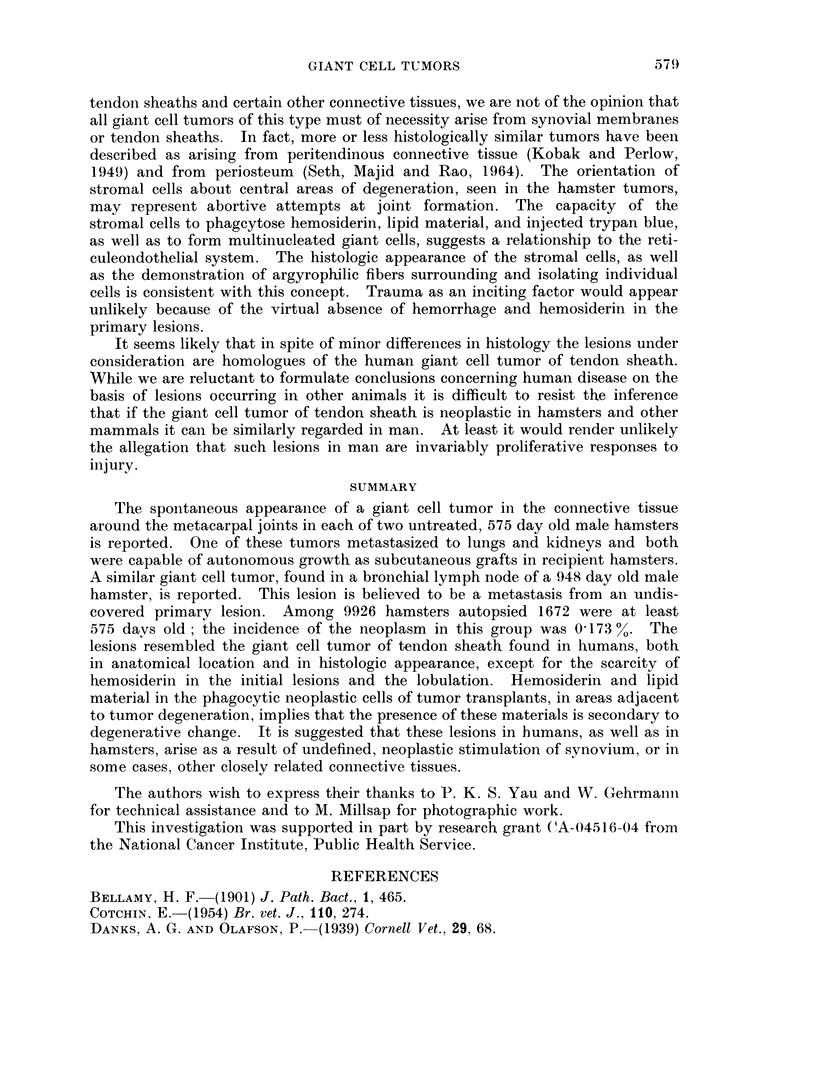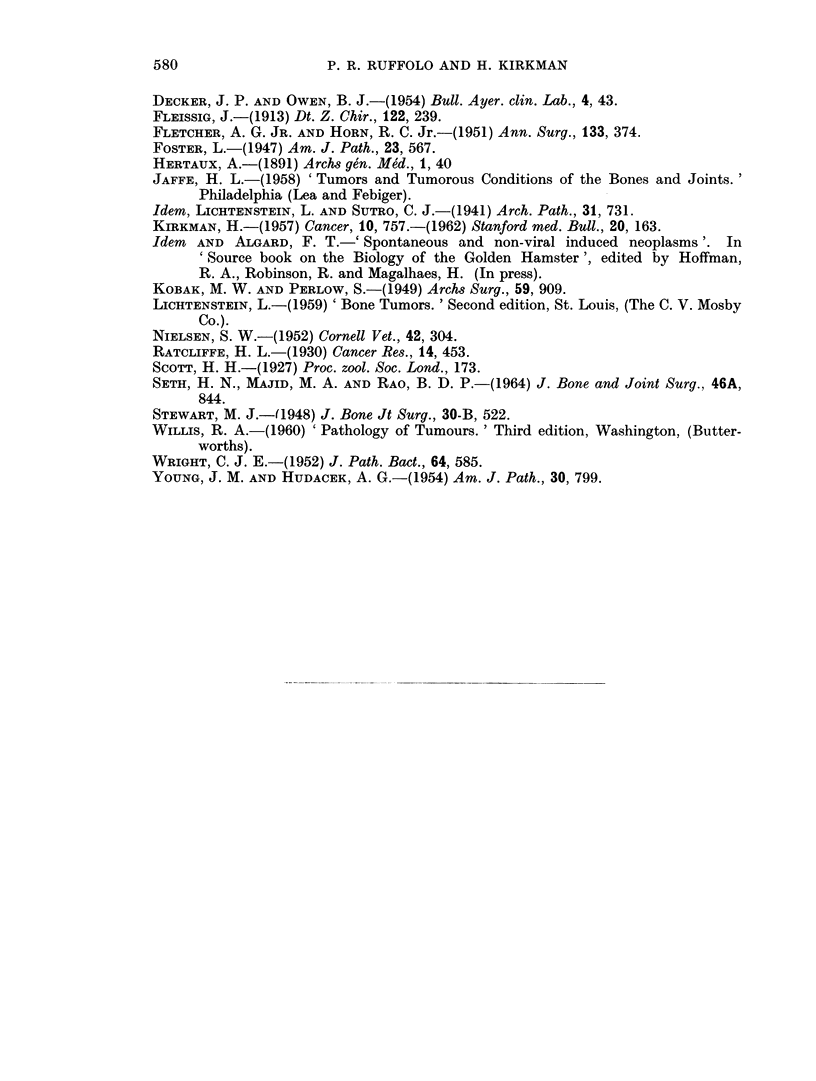# Malignant, transplantable, giant cell tumors of peri-articular connective tissues in Syrian golden hamsters (Mesocricetus auratus).

**DOI:** 10.1038/bjc.1965.71

**Published:** 1965-09

**Authors:** P. R. Ruffolo, H. Kirkman

## Abstract

**Images:**


### MALIGNANT, TRANSPLANTABLE, GIANT CELL TUMORS OF PERI-ARTICULAR CONNECTIVE TISSUES IN SYRIAN GOLDEN HAMSTERS (*MESOCRICETUS AURATUS*)

P. R. RUFFOLO* AND H. KIRKMAN

*From the Departments of Pathology and Anatomy, Stanford University School of Medicine, Stanford, California*

Received for publication April 30, 1965.

THE nosological position of that lesion occurring in and about joint surfaces and designated variously as " xanthoma of the tendon sheath ", " localized villonodular synovitis ", " benign giant cell synovioma ", and " giant cell tumor of tendon sheath " remains disputed. The lesion consists of a proliferation of polyhedral and spindle cells in a fibrous stroma, exhibiting a capacity for phagocytosis of hemosiderin and lipid and a proclivity to form multinucleated giant cells. Opinions differ as to its pathogenesis and biological potential, the real schism occurring in regard to whether or not it is a neoplastic or a reactive process. Some writers (Jaffe, 1958; Jaffe, Lichtenstein and Sutro, 1941) point to the benign behaviour and phagocytic capacity of the component cells in human material, as indicative of a reactive process, while others, noting the rare tendency to recurrence following excision and the occasional occurrence of superficial erosion of adjacent bone (Fletcher and Horn, 1951), have considered it to be a neoplasm with an unpredictable degree of malignant potential. The lesion is not uncommon in human material, but very few well documented cases have been described in domestic animals (Cotchin, 1954; Nielsen, 1952).

During a study of tumors in the Syrian hamster (Kirkman, 1957; 1962; Kirkman and Algard, *in press*) instances of giant cell tumor arising in dense connective tissue around metacarpal joints were encountered. In a preliminary report of hamster tumors (Kirkman, 1962) these were listed, but not discussed, as synoviomas, with an incidence of 0·03 % in 7200 hamsters. Both were transplantable, but only one had metastasized (lungs and kidneys). Since then a third extraskeletal giant cell neoplasm, of unknown origin, has been observed in what was assumed to be an enlarged, discolored, bronchial lymph node. These three lesions resembled anatomically the giant cell tumors of tendon sheath occurring in humans and it was thought that an account of them might be of value in elucidating the nature of that perplexing lesion.

### MATERIAL AND METHODS

All three tumors were found in non-inbred, unrelated, untreated males. The animals were reared apart from each other, but each had contact with one to four cage mates of the same sex and age. All were maintained in cages with wire bottoms, and given water and Purina laboratory chow or Wayne lab-blox *ad*

* Present address: Department of Pathology, Dartmouth University School of Medicine, Hanover, New Hampshire.

*libitum* with greens added twice a week. Room temperatures were kept at about 75° F.

Most tumor tissue was fixed in Bouin's fluid, and embedded in paraffin; although 10 % neutral buffered formalin, 80 % ethyl alcohol, Carnoy's and Helly's fluids were used for special purposes. Routine sections were stained with hematoxylin and eosin or with Mallory azan. Special stains employed were Sudan IV, Prussian blue, mucicarmine, periodic acid Schiff (PAS), Van Gieson, methyl green-pyronin, Foot's silver ammonium carbonate, Gram's stain, acid fast stains and phosphotungstic acid hematoxylin (PTAH).

One host, carrying a fourth serial passage of tumor 7381, received daily subcutaneous injections of 0·5 c.c., per gram of body weight, of freshly prepared 0·5 % trypan blue in 0·9 % sodium chloride solution for seven consecutive days; a biopsy specimen of the transplant was removed on the eighth day, fixed in Bouin's fluid, dehydrated in ethanol and embedded in paraffin. Sections were cut at seven microns and counterstained with either azo carmine or hematoxylin and eosin.

Among 9926 hamsters autopsied to date (April 6, 1965) only these three giant cell tumors were encountered, giving an incidence of 0·030 %. The first two tumors occurred in 575 day old, males, the third in a 948 day old male. The animals autopsied included females as well as males, however, and the majority of these were less than 575 days of age. Since we know of no sound reason for believing that either maleness of lack of treatment were significant factors in the etiology of the neoplasms we feel justified in considering the incidence of the tumors among all hamsters of 575 days or more in age (1155 males and 517 females), irrespective of sex or treatment, as probably indicating the incidence to be expected in this age range in other large hamster colonies; this incidence was 0·173 %. Among the males alone it was 0·259 %. The oldest male autopsied was 1286 days old; the oldest female was 1059 days old. The mean age of the 1675 animals in this group was not computed, but was much greater than 575 days.

### GROSS OBSERVATIONS

Specimen No. 1 (No. 7059): A subcutaneous nodule on the dorsolateral surface of the right metacarpal region was noted by Mr. P. K. S. Yau on October 25, 1961. A biopsy was obtained of the lesion two days later and a 2 mg. portion of the macerated biopsy tissue was injected subcutaneously into each of three control hamsters, all which died 211 days later. Transplant growth was detected in one of the three. This transplant was palpable after 125 days and at autopsy weighed 1674 mg., or 1·70 g. per 100 g. body weight, and showed a mean daily growth rate of 8 mg. The tumor was retransplanted into three hosts, but, due to advanced post mortem changes in the donor tissue, did not grow. Grossly, the neoplasm was moderately soft, well circumscribed, and grey in color. There were no yellow or brown regions suggesting lipid or hemosiderin-containing areas. The original donor animal died at 721 days of age. By this time the tumor mass had increased in diameter from 11 to 18 mm. and weighed 1995 mg. or 2·53 g. per 100 g. body weight. It had a mean daily growth rate of 14 mg. Grossly, it did not invade bone, but was firmly adherent to the peri-articular connective tissue, was discrete and unattached to the overlying skin. The transected surface disclosed a firm, grey tumor of uniform texture well delineated by the compressed adjacent connective tissue. Lobulation was not conspicuous. Examination of the lungs disclosed a number of grey-white metastatic nodules, measuring from 1 to 3 mm. in diameter, distributed irregularly in both lungs. A single minute pale area in the left kidney, and two similar ones in the right, proved to be metastases. Lymph nodes, spleen and liver were of normal weight and free of lesions, as were the thyroid and parathyroid glands. The rest of the autopsy was similarly unremarkable, except for a medium-sized cortical adenoma in each adrenal gland.

Specimen No. 2 (No. 7381) : A firm tumor on the lateral margin of the left metacarpal area was noted in this animal by Mr. Yau on July 17, 1962. No metastases, additional neoplasms, or other lesions were found. The mass was not weighed, but measured 4 mm. in diameter, was firm, bluish-grey on the transected surface, and firmly adherent to peri-articular connective tissue, showing no infiltration of bone or attachment to overlying skin. Biopsies of 2 mg. each from the lesion were transplanted to the subcutaneum of two control hamsters. The remainder of the tumor was preserved for histological examination. The transplant became palpable in one of the recipients after 64 days. The host survived for 238 days, during which time the transplant attained a weight of 10 g., or 12·8 g. per 100 g. body weight, and showed a mean daily growth rate of 42 mg. Tissue taken from the transplant was used as a homograft in two other hamsters and grew in each. The tumor is now in the fourteenth passage.

Specimen No. 3 (No. 9286) : This animal was found dead on April 26, 1964. At autopsy a large left adrenal adenocarcinoma was found. The right cervical and left bronchial lymph nodes were enlarged and appeared to be hemorrhagic. All liver lobes contained large clusters of clear vesicles of intrahepatic bile duct origin. The testes were atrophic, but the accessory organs of reproduction were of normal size and appearance. There was a moderate amount of subcutaneous, and a marked amount of pulmonary, edema. Some hemorrhagic ascites was present. No giant cell tumor was detected until microscopic examination of what had been supposed to be an enlarged superior tracheobronchial lymph node was made.

### HISTOLOGIC OBSERVATIONS

Specimen No. 1 : The biopsy tissue disclosed a lesion consisting of several types of cells (Fig. 1). The predominant one was a large, spherical to spindle-shaped, stromal cell with a moderately abundant, eosinophilic cytoplasm. Nuclei were ovoid, regular in contour, with a moderately dense to vesicular chromatin pattern. Occasional nuclei showed irregular chromatin condensation both at the periphery of the nuclear membrane and in the central portion. Nucleoli were present but small. Mitotic figures were seen only occasionally. Another cell type was more fusiform, with a somewhat denser nucleus and tapering eosinophilic cytoplasm ; it closely resembled a fibroblast. Mutinucleated, tumor giant cells with nuclei resembling those of both stromal types were numerous. Many of these contained centrally placed nuclei, but larger giant cells of the Langhans type, with peripherally arranged nuclei, were abundant. The overall histological appearance was that of an essentially benign neoplastic growth with an eosinophilic fibrous stroma of variable density. There were occasional clusters of inflammatory polymorphonuclear and lymphocytic cells in areas of degeneration. Iron stains revealed infrequent hemophages, but foam cells were present in occasional clusters. The giant cells contained neither stainable pigment nor foreign material. Clefts, lined by more or less cuboidal occurred (Fig. 2) but were rare. Thin-walled blood vessels were fairly prominent toward the central portion of the lesion. Gram stains and stains for acid-fast bacilli were negative.

Autopsy specimens had essentially the same histologic appearance. One notable change was the less frequent occurrence of giant cells and an increased cellularity with packed polyhedral stromal cells comprising the bulk of the growth. In a few areas the fusiform stromal cells were arranged in a palisaded fashion about central areas of degeneration (Fig. 3). The latter contained an amorphorous, faintly eosinophilic material which did not stain by any of the methods employed for mucin or glycogen. There was no evidence of the formation of epithelial knobs on a dense stroma, as seen in malignant synovioma, nor was the formation of slit-like spaces and clefts by a trabecular arrangement of tumor cells a prominent feature. Tumor cells showed an apparent increase in the nucleo-cytoplasmic ratio, more division figures, and an increase in nuclear density and pleomorphism. Some stromal cells with their distinct cytoplasm, round, central or eccentric, vesicular nuclei and their tendency to occur in sheets, were suggestive of reticulum cells. Others resembled the plasma-cell series (Fig. 4). However, they were not pyroninophilic and showed no distinct perinuclear " halos ". There were numerous binucleate and tetranucleate forms.

The metastatic lesions presented the same histologic features as the primary ones. In the lungs (Fig. 5) they were situated close to the walls of bronchioles. Smaller nodules were apposed to dilated vascular channels, but bore no obvious connection to the endothelial cells. The small renal metastases were all cortical (Fig. 6). PAS and Gram stains as well as stains for acid-fast organisms were negative.

### EXPLANATION OF PLATES

FIG. 1.—A typical area in a section through a primary giant cell tumor of periarticular connective tissue (No. 7059). It demonstrates multinucleated giant cells and spindle and ovoid shaped cellular components of the neoplasm. H. and E. ×600.

FIG. 2.—A small cleft partially lined by more or less cuboidal tumor cells simulating, but not constituting, an epithelium in a primary tumor (No. 7059). H. and E. ×600.

FIG. 3.—(An area of tumor cells exhibiting palisading about areas of stromal degeneration, possibly expressing the embryonic potential for the formation of joint cavities (No. 7059). H. and E. ×750.

FIG. 4.—An area of primary neoplasm (No. 7059) containing cells resembling reticulum cells, plasma cells and intermediate forms between them, as well as giant cells. Mallory azan stain. ×375.

FIG. 5.—One of numerous pulmonary metastases from a primary giant cell tumor (No. 7059). H. and E. ×375.

FIG. 6.—A renal cortical metastasis from the same tumor (No. 7059). H. and E. ×900.

FIG. 7.—A typical area in the first serial subpannicular transplant of neoplasm No. 7059. H. and E. ×600.

FIG. 8.—Reticular fibers isolating tumor cells individually and in small clusters in a fourth generation subpannicular transplant from tumor No. 7381. Silver ammonium carbonate stain. ×600.

FIG. 9.—A typical area in a lymph node metastasis of a presumed giant cell tumor of tendon sheath (No. 9286) from an undiscovered primary lesion. Illustrated are a subcapsular zone of foam cells and numerous multinucleated giant cells in a background composed of spindle and polyhedral shaped cells in a collagenous stroma. Areas of loosely arranged cells suggesting abortive attempts at synovial cavity formation are present. H. and E. ×140.

Ruffolo and Kirkman.

Ruffolo and Kirkman.

Ruffolo and Kirkman.

The successful homograft (Fig. 7) disclosed essentially the same histologic features as did the primary and metastatic lesions. Giant cell formation was reduced in the young transplant and the stromal cells were predominant, many displaying a granular, rounded to fusiform cytoplasm. The vascularity increased and there were numerous blood vessels of capillary size within the central portion of the neoplasm.

Specimen No. 2 : The histopathology was very similar to that of the first tumor and the cytoplasm of the stromal cells had a faintly granular, homogeneous appearance again bearing a resemblance to reticulum cells. Finely vacuolated, stromal cells, recognizable as lipid-containing histiocytes, were more numerous in this neoplasm. Stains for mucin, glycogen and iron were negative. There were some areas of simple necrosis at the edges of which a fusiform type of neoplastic cell was again palisaded. This feature was similar to, but less prominent, than in Specimen No. 1. The number of giant cells was not as striking as in the first case, but when present, their nuclei tended to exhibit a slightly greater degree of atypicality. Cross striations were not found in sections stained by the PTAH method ; there were no areas of gland-like, papilliferous, epithelial proliferation, or cleft formation to suggest a malignant synovioma.

The transplanted tumor was lobulated, poorly defined, and gave gross evidence of infiltration into the underlying skeletal muscle. The transected surface was soft and grey-white in color.

The histological appearance was essentially the same in all cases, bearing a striking resemblance to the original tumors, except for some increase in anaplasia and an increased frequency of mitoses. In general, the multinucleated giant cells were inconspicuous during early growth of transplants, becoming larger, more abundant, and acquiring more nuclei, as the transplants aged and enlarged. There were occasional areas of tumour degeneration and section taken from these areas contained minimal deposits of fine, particulate, Prussian-blue positive material. Fat stains disclosed fine lipid particles in some of the tumor cells. Some of the plasma cells adjacent to areas of degeneration were pyroninophilic, but none of the neoplastic cells displayed this characteristic. The overall cellularity, nuclear atypia and evidence of skeletal infiltration were evidence of the continued malignancy of this transplanted neoplasm. Reticular fiber stains disclosed a pattern in which the proliferating neoplastic cells were surrounded both individually and in small clusters (Fig. 8).

Transplant sections from a trypan blue injected host demonstrated some capacity, on the part of the multinucleated giant cells, and some of the typical mononucleated tumor cells, to phagocytose the dye.

Specimen No. 3 : This lesion is interpreted as a metastasis from an unknown primary. Although presumed to be in a lymph node, on the basis of gross examination, no recognizable nodal tissue or pericapsular lymphatic vessels were found. The ovoid, encapsulated mass consisted of a rather sparse collagen fiber stroma, interdigitating bundles of spindle-shaped tumor cells, many foam cells and abundant multinucleated giant cells, including many of the Langhans type, some of which had conspicuously vacuolated cytoplasm and other indications of phagocytic activities. Scattered areas of loosely arranged cells suggestive of abortive attempts at synovial cavity formation were present. The histology was essentially similar to that of the two primary lesions and their metastases and transplants (Fig. 9).

### DISCUSSION

A few other isolated examples of extraskeletal neoplastic giant cell lesions, probably of tendon sheath origin, have been observed in animals other than man. Six of these were reported in dogs by Cotchin (1954) ; one of the six had metastasized to the lungs. A similar one described as malignant, but in which no metastases were found, was reported by Nielsen (1952) in a cat. None of the giant cell tumors described by Danks and Olafson (1939) in a mule, by Scott (1927) in an Indian rhinoceros, and by Ratcliffe (1930) in a baboon, appear to have been associated with bone involvement and may have been of tendon sheath origin, but data are too few to permit final decisions.

Although the giant cell tumor of tendon sheath was frequently presumed to be a sarcoma during the nineteenth century, it was described as a benign lesion by Hertaux in 1891. Fleissig (1913) considered it to be inflammatory and it was regarded as an angioblastic endothelioma by Bellamy (1901). More recently a similar position was taken by Foster (1947) who considered it to be a type of sclerosing hemangioma. Jaffe *et al.* (1941) maintained that the tumor was a localized nodular form in a spectrum of inflammatory synovial hyperplasias which they called villonodular synovitis ; Lichtenstein (1959) reiterated this position recently. These authors called attention particularly to the fusion of adjacent hyperplastic villi as the mechanism of formation of the solitary nodule. Young and Hudacek (1954), in experimental attempts to produce the lesion by repeated intratendinous injections of autologous blood in dogs, produced diffuse articular lesions with fusion of enlarged papilliferous synovial nodules containing hemophages and foam cells in great number in a fibrous stroma, but the solitary form of the disease has not been produced in experimental animals. In the hamster tumors there was no evidence of fusion of adjacent villi, with consequent lobulation to indicate an origin from reactive synovitis. Hemorrhage appeared to play no role in the pathogenesis of the lesions and what little intracellular hemosiderin was found was secondary to retrogressive changes.

Wright (1952), Stewart (1948), Willis (1960), and others regarded such lesions as neoplasms in humans and in Wright's series of forty-seven synoviomas two were designated malignant variants of the benign giant cell tumor of the tendon sheath. One of these recurred locally and showed evidence of skeletal muscle infiltration. The other eventually metastasized. Decker and Owen (1954) reported a well documented metastasizing case. Kobak and Perlow (1949) described another with widespread metastases.

The ability to metastasize, and transplantability as an autonomous homologous graft are sufficient indications that the hamster lesion is a true malignant neoplasm. The architectural features of neoplasms arising primarily from blood vessels, fibroblasts, skeletal muscle or bone are lacking in the hamster lesions. In our colony no bone tumors have been found. No other tumors have occurred which appear sufficiently to resemble the three described in this report to be grouped with them. These include five fibromas, ten fibroangiomas, eleven hemangiomas, one hemangiopericytoma, eleven neurofibromas, two neurofibrosarcomas and one leiomyosarcoma (plus many induced leiomyosarcomas). On the basis of anatomic location, histology and possession of certain microscopic features of synovial membranes the latter structures are considered to be the probable sites of origin. Since the histological components of synovial membranes are shared by tendon sheaths and certain other connective tissues, we are not of the opinion that all giant cell tumors of this type must of necessity arise from synovial membranes or tendon sheaths. In fact, more or less histologically similar tumors have been described as arising from peritendinous connective tissue (Kobak and Perlow, 1949) and from periosteum (Seth, Majid and Rao, 1964). The orientation of stromal cells about central areas of degeneration, seen in the hamster tumors, may represent abortive attempts at joint formation. The capacity of the stromal cells to phagcytose hemosiderin, lipid material, and injected trypan blue, as well as to form multinucleated giant cells, suggests a relationship to the reticuleondothelial system. The histologic appearance of the stromal cells, as well as the demonstration of argyrophilic fibers surrounding and isolating individual cells is consistent with this concept. Trauma as an inciting factor would appear unlikely because of the virtual absence of hemorrhage and hemosiderin in the primary lesions.

It seems likely that in spite of minor differences in histology the lesions under consideration are homologues of the human giant cell tumor of tendon sheath. While we are reluctant to formulate conclusions concerning human disease on the basis of lesions occurring in other animals it is difficult to resist the inference that if the giant cell tumor of tendon sheath is neoplastic in hamsters and other mammals it can be similarly regarded in man. At least it would render unlikely the allegation that such lesions in man are invariably proliferative responses to injury.

### SUMMARY

The spontaneous appearance of a giant cell tumor in the connective tissue around the metacarpal joints in each of two untreated, 575 day old male hamsters is reported. One of these tumors metastasized to lungs and kidneys and both were capable of autonomous growth as subcutaneous grafts in recipient hamsters. A similar giant cell tumor, found in a bronchial lymph node of a 948 day old male hamster, is reported. This lesion is believed to be a metastasis from an undiscovered primary lesion. Among 9926 hamsters autopsied 1672 were at least 575 days old ; the incidence of the neoplasm in this group was 0·173 %. The lesions resembled the giant cell tumor of tendon sheath found in humans, both in anatomical location and in histologic appearance, except for the scarcity of hemosiderin in the initial lesions and the lobulation. Hemosiderin and lipid material in the phagocytic neoplastic cells of tumor transplants, in areas adjacent to tumor degeneration, implies that the presence of these materials is secondary to degenerative change. It is suggested that these lesions in humans, as well as in hamsters, arise as a result of undefined, neoplastic stimulation of synovium, or in some cases, other closely related connective tissues.

The authors wish to express their thanks to P. K. S. Yau and W. Gehrmann for technical assistance and to M. Millsap for photographic work.

This investigation was supported in part by research grant CA-04516-04 from the National Cancer Institute, Public Health Service.

### REFERENCES

Bellamy, H. F.—(1901) *J. Path. Bact.*, **1**, 465.
Cotchin, E.—(1954) *Br. vet. J.*, **110**, 274.
Danks, A. G. and Olafson, P.—(1939) *Cornell Vet.*, **29**, 68.

DECKER, J. P. AND OWEN, B. J.—(1954) *Bull. Ayer. clin. Lab.*, **4**, 43.
FLEISSIG, J.—(1913) *Dt. Z. Chir.*, **122**, 239.
FLETCHER, A. G. JR. AND HORN, R. C. Jr.—(1951) *Ann. Surg.*, **133**, 374.
FOSTER, L.—(1947) *Am. J. Path.*, **23**, 567.
HERTAUX, A.—(1891) *Archs gén. Méd.*, **1**, 40
JAFFE, H. L.—(1958) 'Tumors and Tumorous Conditions of the Bones and Joints.' Philadelphia (Lea and Febiger).
*Idem*, LICHTENSTEIN, L. AND SUTRO, C. J.—(1941) *Arch. Path.*, **31**, 731.
KIRKMAN, H.—(1957) *Cancer*, **10**, 757.—(1962) *Stanford med. Bull.*, **20**, 163.
*Idem* AND ALGARD, F. T.—'Spontaneous and non-viral induced neoplasms'. In 'Source book on the Biology of the Golden Hamster', edited by Hoffman, R. A., Robinson, R. and Magalhaes, H. (In press).
KOBAK, M. W. AND PERLOW, S.—(1949) *Archs Surg.*, **59**, 909.
LICHTENSTEIN, L.—(1959) 'Bone Tumors.' Second edition, St. Louis, (The C. V. Mosby Co.).
NIELSEN, S. W.—(1952) *Cornell Vet.*, **42**, 304.
RATCLIFFE, H. L.—(1930) *Cancer Res.*, **14**, 453.
SCOTT, H. H.—(1927) *Proc. zool. Soc. Lond.*, 173.
SETH, H. N., MAJID, M. A. AND RAO, B. D. P.—(1964) *J. Bone and Joint Surg.*, **46A**, 844.
STEWART, M. J.—(1948) *J. Bone Jt Surg.*, **30**-B, 522.
WILLIS, R. A.—(1960) 'Pathology of Tumours.' Third edition, Washington, (Butterworths).
WRIGHT, C. J. E.—(1952) *J. Path. Bact.*, **64**, 585.
YOUNG, J. M. AND HUDACEK, A. G.—(1954) *Am. J. Path.*, **30**, 799.